# Reduction of cadmium toxicity in wheat through plasma technology

**DOI:** 10.1371/journal.pone.0214509

**Published:** 2019-04-01

**Authors:** Ahmad Humayan Kabir, Md Mosiur Rahman, Urmi Das, Urmi Sarkar, Nepal Chandra Roy, Md Abu Reza, Mamunur Rashid Talukder, Md Alfaz Uddin

**Affiliations:** 1 Molecular Plant Physiology Laboratory, Department of Botany, University of Rajshahi, Rajshahi, Bangladesh; 2 Plasma Science and Technology Laboratory, Department of Applied Physics and Electronic Engineering, University of Rajshahi, Rajshahi, Bangladesh; 3 Molecular Biology and Protein Science Laboratory, Department of Genetic Engineering and Biotechnology, University of Rajshahi, Rajshahi, Bangladesh; 4 Department of Physics, University of Rajshahi, Rajshahi, Bangladesh; Universidade de Coimbra, PORTUGAL

## Abstract

Cadmium (Cd) contamination in plant-derived food is a big concern. This study examines whether and how Ar/O_2_ and Ar/Air plasma techniques lead to Cd detoxification in wheat. Treatment with Ar/O_2_ and Ar/Air changed the seed surface and decreased the pH of seeds as well as the cultivation media. Generally, plants subjected to Cd treatment from seeds treated with Ar/O_2_and Ar/Air plasma showed considerable progress in morphology and total chlorophyll synthesis compared to Cd-treated wheat, suggesting that plasma technology is effective for Cd detoxification. Furthermore, Ar/O_2_ and Ar/Air plasma treated plants showed a significant decrease in root and shoot Cd concentration, which is consistent with the reduced expression of Cd transporters in the root (*TaLCT1* and *TaHMA2*) compared with the plants not treated with plasma in response to Cd stress. This Cd inhibition is possibly accomplished by the decrease of pH reducing the bioavailability of Cd in the rhizosphere. These observations are in line with maintenance of total soluble protein along with reduced electrolyte leakage and cell death (%) in root and shoot due to Ar/O_2_ and Ar/Air treatments. Further, Cd-induced elevated H_2_O_2_ or oxidative damage in tissues was mainly diminished through the upregulation of antioxidant enzymes (SOD and CAT) and their corresponding genes (*TaSOD* and *TaCAT*) induced by Ar/O_2_ and Ar/Air plasma. Grafting results suggest that root originating nitric oxide signal possibly drives the mechanisms of Cd detoxification due to plasma treatment in wheat. These findings provide a novel and eco-friendly use of plasma technology for the mitigation of Cd toxicity in wheat plants.

## 1 Introduction

Wheat (*Triticum aestivum* L.) is an important cereal crop in many countries. The production of wheat under threatfrom climatic (temperature, humidity, and drought, etc) and heavy metal contamination [1,2]. Furthermore, unplanned industrialization and agro-chemicals can increase the concentration of toxic metals in soil and other ecosystems. Excess Cd causes chlorosis, wilting, leaf roll, and growth retardation in plants [3–5]. In addition, Cd toxicity may reduce photosynthesis, gas exchange characteristics, membrane stability, protein synthesis, stomatal conductance, and transpiration rate in plants [6–7]. These physiological and morphological disorders eventually cause the low production of wheat which is in high demand for the increased population. In plants, the pH surrounding rhizosphere is pivotal for the bioavailability of nutrients and metal elements [8]. Further, the soil pH influences the capacity and transfer of Cd in plants [9]. Cd is transported to plant cell though carriers via active and passive pathways [10–11]. It was previously shown that low-affinity cation transporter (*LCT1*) and heavy metal ATPase *(HMA2*) showed elevated expression subjected to Cd stress in wheat [10].

A Cd-free food supply is of the utmost importance for human health. To ensure this, detoxification of industrial effluents or elimination of toxicity is a big challenge. More importantly, strategies are currently focused on environmentally friendly technologies. Very recently, plasma technology has been proven to play a role in agricultural improvement and tolerance of abiotic stress in a few crop species [11–12]. Due to its ability to induce germination and growth without chemicals, plasma treatment attracts a lot of attention. Due to its stability and high volume compared to the atmospheric pressure plasma source, the low- pressure dielectric barrier discharge (LPDBD) technique could be an important supply of non-thermal plasma. Its potential impacts on the energy conversion, environment, biology and sterilization are also applicable [13–15].

Seed germination and growth are linked to several physiological and biochemical indicators in plants. Hydrogen peroxide (H_2_O_2_) is a reactive molecule responsible for both negative and positive roles in plant physiological processes together with stress tolerance. The roles of H_2_O_2_ in plant systems depend on the physiological conditions, concentrations and types (heavy metal, mineral deficiency, drought etc.) of the stresses [16–17]. H_2_O_2_ is also regarded as a signaling hub for the regulation of seed dormancy, germination and antioxidant defense regulation [16]. In addition, rhizospheric acidification due to the secretion of the proton (H^+^) causes low pH. This mechanism is crucial for plants to acquire minerals or nutrients. Absorption of Cd was reduced owing to acidification and increased competition with hydrogen ion in plant species [17]. Further, the plasma discharge may decrease the pH up to five times [15].

It was clear that plasma stimulates germination of seeds and wheat growth [18–19]. The cutback of rhizosphere Cd bioavailability is one of the mechanisms that may lessen Cd in plants. The pH of the rhizosphere affects a plant's capacity to uptake Cd [20]. However, none of the phytoremediation techniques are fully efficient and eco-friendly. Moreover, the application of plasma technology in Cd detoxification in crop plants has not yet been reported. We have therefore performed a series of physiological, biochemical and molecular experiments to investigate the role, if any, of LPDBD to limit Cd-induced phytotoxicity in wheat plants.

## 2 Materials and methods

### 2.1 Production of plasma and identification of species

Plasma production and species identifications were performed as previously described [19]. Briefly, copper electrode was placed at the lower end of a test tube and another one was covered by a glass tube containing wheat seeds attached to the upper end of the discharge tube (S1 Fig). Plasma at the pressure ~10 torrs was generated by high voltage (5–10 kV, 3–8 kHz) bipolar sinusoidal power supply to the electrode. The flow of Ar, O_2,_ and Air in the chamber was maintained by three different gas flow meters Yamato, KIT and 115P, respectively. The amount of power absorbed by the plasma was ~45 W for Ar/ Air gas mixture measured by applying voltage 5 kV of frequency 4.5 kHz with the spacing of electrode being 60 mm.

The plasma-induced emission spectra were recorded with the spectrometer AvaSpec-2018 (USB2000+XR1, slit size: 25μm, grating: 800 lines/mm, optical resolution: 1.7 nm, wavelength range: 200-1100nm, slit: 10μm, gratting: 2400 lines/mm, optical resolution: 0.07 nm, wavelength range: 200-500nm). The ROS (reactive oxygen species) and RNS (reactive nitrogen species) produced were Ar/O_2_ (Ar: 60%, O_2_: 40%) and Ar/Air (Ar: 60%, O_2_: 40%) at voltage: 5kV, electrodes spacing: 40 mm, pressure (~10 Torr) (S1 Fig). Production of ROS and RNS depend on the gas molecules or atoms presented inthe discharge region but their percentage is the same. Species identification is clearly mentioned in the supplementary S1C Fig. The flow rates of Ar, O_2,_ and Air are 0.6 L/min, 0.4L/min and 0.4 L/min respectively and these rates were controlled by three different gas flow meters Yamato, KIT and 115 P respectively.

The wavelength of N^+^ atomic transitions was 517.52 nm and 668.22 nm. Further, transitions of O radicals occurred at 777.1 and 844.2 nm for Ar/O_2_ plasma. ROS has also been observed as O^+^ and O_2_^+^ ionic transition. The NO band transition ($A^{2}\Sigma^{+}\to X^{2}\Pi )$ was found in Ar/O_2_ plasma shown in S1 Fig. The transition of Ar lines was detected at 685–914 nm due to the presence of Ar gas. In addition, the transition of $H_{\beta }$ and $H_{\alpha }$ lines were found both for Ar/Air and Ar/O_2_ plasmas. Rotational ($T_{rot})$ and vibrational ($T_{vib})$ temperatures were determined through a stimulated negative system ${\text{N}}_{2}^{+}\left({\text{B}}^{2}\Sigma_{\text{u}}^{+}-{\text{X}}^{2}\Sigma_{\text{g}}^{+}\right)$, using LIFBASE spectroscopic software. The wheat seeds were selected randomly both for treatment and control [19].

### 2.2 Treatment of seed and cultivation of plants

LPDBD plasma treated the wheat seeds (BARI Gom 22) with the following gas mixtures: Ar/Air and Ar/O_2_. The gas mixture temperature was ~304 K and measured by a thermometer at 2 mm from the electrodes. The seed treatment chamber was made by a cylindrical glass tube. The height of the tube is 90 mm and inside diameter is 15mm. A maximum of 20g seeds can be treated at one time in a seed chamber. The upper side was open while a cotton mesh covered the lower side. The seeds were arranged to have optimum surface treatment for 90 s. Plasma treatments did not introduce temperature change as the gas temperature was fixed at the room temperature 304 K. For quality control, the control seeds were placed on the chamber but not treated with plasma. In this study, 10g seeds were used for control and treatments.

Plasma-treated seeds were germinated on moist Petri dishes prior to hydroponic culture[21] containingthe following elements (μM):KNO_3_ (16000), Ca(NO_3_)_2_.4H_2_O (6000), NH_4_H_2_PO_4_ (1000), MgSO_4_.7H_2_O (2000), KCl (50), H_3_BO_3_ (25), Fe-EDTA (25), MnSO_4_.4H_2_O (2), ZnSO_4_ (2), Na_2_MoO_4_.2H_2_O (0.5) and CuSO_4_.5H_2_O (0.5). Cd stress was induced [22] by adding 10 μM CdSO_4_ to the solution culture (pH 6.0). In the controlled growth cabinet (25°C and 70% humidity), wheat seedlings were grown in 2 L plastic pot under 14 h dark and 10 h light intensity (550–560 μmol s^-1^ per μA). Plants were grown for 7d once transferred to solution culture and then harvested.

### 2.3 Morphological features, chlorophyll (*a* and *b*) and pH determination

Once harvested, the length of the root and shoot was manually measured using a digital caliper. Separated roots were then washed with deionized water and twice blotted in tissue papers. These plant tissues were further dried in an electric oven for 2 d at 80°C before their dry weight was measured. The total concentration of chlorophyll (*a* and *b*) in young leaves was determined as previously described [23]. Briefly, young fresh leaves (10 mg) were homogenized in 90% methanol using mortar and pestle. The samples were then centrifuged for 5 min at 12000 rpm and the cell debris was discarded. Finally, a spectrophotometer (UV-1650PC, Shimadzu) recorded the absorption of the clear supernatant at 662 nm (chlorophyll *a*) and 646 nm (chlorophyll *b*). The optical density of clear supernatant was monitored at 662 nm and 646 nm by UV-1650PC spectrophotometer (Shimadzu). The concentration of chlorophyll *a* and *b* werecalculated as previously described [23]. In addition, the pH of the cultivation solution was directly measured using a Horiba LAQUA twin Compact pH Meter (Japan). For seed pH analysis, seeds were squeezed out a few drops of sap using a stainless garlic press. The sap was then placed on the sensor of the pH meter and further measured.

### 2.4 Determination of Cd in root and shoot

Once harvested, the root and shoot were placed in 1.5 ml Eppendorf tube. Additionally, roots were washed in CaSO_4_ (1 mM) for 5 minin order to remove contaminants from the surface. Samples were cleaned with deionized water 2–3 times before drying at 80°C for 2d in the oven with deionized water. Samples (0.5 g) were then digested in a glass beaker with 5 ml HNO_3_ and 2 ml HClO_4_ and heated in a microwave oven. The standard solutions of Cd were independently prepared and further diluted for the preparation of the calibration curve. The Cd concentration was then determined by Flame Atomic Absorption Spectroscopy (Model No. AA-6800, Shimadzu) as previously described [3].

### 2.5 Estimation of total soluble protein

Total soluble protein was determined as previously described [24]. Briefly, clean plant tissue (10 mg) was homogenized with mortar and pestle containing 50 mM Tris-HCl (pH 7.5), 2 mM EDTA (ethylenediaminetetraacetic acid) and 0.04% (v/v) 2-mercaptoethanol. The mixture was then centrifuged at 12000 rpm at 25°C for 10 min before the cell debris was discarded. The supernatants were then mixed with 1 ml Coomassie Brilliant Blue (5 μg/ml) and monitored at 595 nm in a spectrophotometer. Finally, a standard curve of bovine serum albumin (BSA) was plotted for calculating total soluble protein.

### 2.6 Measurement of electrolyte leakage

The electrolyte leakage, anindicator of membrane damage, was measured in both root and shoot by a digital electrical conductivity meter [25]. To eliminate surface elements, plant roots and shoots were washed with deionized water. Afterward, the samples were incubated in a vial containing 20 ml of deionized waterand shaken for 2 h at room temperature. Finally, the solution's electrical conductivity was recorded.

### 2.7 Determination of cell death

Analysis of cell death was carried out with some modifications following the Evans blue method [26]. At room temperature, root and shoot were initially incubated in 0.25% Evans blue solution for 15 min at room temperature. The solution was subsequently replaced by 1 ml of 80% ethyl alcohol for 10 min. The samples were then incubated at 50°C for 15min in a water bath. In addition, the samples were centrifuged for 10 min at 12,000 rpm. The supernatant's optical density was finally recorded at 600 nm. Finally, cell death was calculated on the basis of the fresh weight of tissue used.

### 2.8 Determination of H_2_O_2_

The root and shoot (10 mg) were homogenized in 1ml of 0.1% trichloroacetic acid (TCA) for H_2_O_2_ analysis. [27]. The sample mixture was centrifuged for 15 min at 10,000 rpm before the cell debris was discarded. The clear supernatant was then added potassium iodide (1 M) and phosphate buffer (10 mM, pH 7.0) and kept in the dark for 1 h. Finally, a spectrophotometer (UV-1650PC, Shimadzu) was used to measure the optical density of the extract mixture at 390 nm.

### 2.9 Analysis of antioxidant enzymes (SOD, APX, and CAT)

In the first place, tissues (10 mg) were ground using mortar and pestle in 1 ml phosphate buffer (100 mM, pH 7.0). The homogenate was then centrifuged for 10 min at 8000 rpm) and separated into new tubes [28]. For the analysis of superoxide dismutase (SOD), the plant extracts (100μl) were added to assay solution containing 0.1 mM EDTA, 50 mM sodium bicarbonate (pH 9.8) and 0.6 mM epinephrine [29]. After 4 min, the formation of adrenochrome was read at 475 nm in a UV-Vis spectrophotometer. Ascorbate peroxidase (APX) activity was analysed in a reaction mixture supplemented with 0.1 mM EDTA, 50 mM potassium phosphate (pH 7.0), 0.1 mM H_2_O_2_, 0.5 mM ascorbic acid, and 0.1 ml extract. The calculation for APX activity was performed on the basis of the extinction coefficient (2.8 mM^-1^ cm^-1^) following the optical density of the assay mixture taken at 290 nm [3]. The absorbance of catalase (CAT) in the reaction mixture with100 mM potassium phosphate buffer (pH 7.0), 6% H_2_O_2_ and 100 μl root extract was read at 240 nm (extinction coefficient of 0.036 mM^−1^ cm^−1^) using a UV spectrophotometer at 30s intervals up to 1 min.

### 2.10 Analysis of nitric oxide (NO) in root and shoot

NO was analyzed on the basis of hemoglobin absorbance as a consequence of its conversion from oxyhemoglobin (HbO_2_) to methemoglobin (metHb) in the presence of NO [19]. In 1 ml of cooled assay buffer containing 0.1 M sodium acetate, 1 M NaCl and 1% (w/v) ascorbic acid (pH 6.0), the harvested root or shoot samples were grounded. The homogenates were centrifuged at 10,000 rpm at 4°C for 5 min, and the supernatants were transferred to a centrifuge tube. The HbO_2_ solution stock (5 mM) was subsequently added to the samples and incubated at room temperature for 5 min. The conversion rate of HbO_2_ to metHb was assessed at 401 nm.

### 2.11 RNA isolation and gene expression analysis

The expression of *TaLCT1*, *TaHMA2*, *TaSOD*, *TaAPX*, and *TaCAT* transcripts was performed in roots by real-time PCR (reverse transcription PCR). Firstly, roots (50–70 mg) were homogenized to a fine powder by mean of liquid nitrogen in a chilled mortar and pestle. The total RNA was subsequently extracted in accordance with the SV Total RNA Isolation System protocol (Cat. No. Z3100, Promega Corporation, USA). RNA samples were subsequently checked for superiority by denaturing gel electrophoresis. Once UV-Vis Spectrophotometer (NanoDrop 2000) quantified the RNA, the first-strand cDNA was synthesized according to the instructions of the GoScriptTM Reverse Transcription System (Cat No. A5001, Promega Corporation, USA). The cDNA samples subsequently incubated with RNase enzymes to remove contamination with RNA. The real-time PCR analysis was performed in Eco^TM^ real-time PCR system controlled by Eco Software v4.0.7.0 (Illumina, USA). Nucleotide sequences of each gene-specific primer were presented in Supplementary S1 Table. The expression data were normalized with *Actin* as an internal control (Eco Software v4.0.7.0).The real-time PCR program used was as follows: 3 min at 95°C,40 cycles of 30 s at94°C,15 s at56°C and 30 s at72°C.

### 2.12 Grafting of control and Ar/O_2_ treated seedlings

On young seedlings after germination, reciprocal grafting between control and Ar/O_2_ treated plants was carried out. Newly emerging plants treated with or without Ar/O_2_ were cut diagonally on small stems (45 from the horizontal) 0.4 cm above the seeds. Scion (the portion removed) detached were grafted onto rootstocks in four different combinations (type 1: control self-grafting, type 2: Ar/O_2_ self-grafting, type 3: control rootstock + Ar/O_2_ scion, Type 4: Ar/O_2_ rootstock + control scion). A thin capillary tube placed over the graft held each graft together and then transferred to the hydroponic conditions. Grafted plants with adventitious rooting were discarded in the following days.

### 2.13 Statistical analysis

All experiments had three independent biological replications for each sample. Statistical analyses were performed at a 5% significance level by two-way ANOVA followed by Duncan's Multiple Range Test (P< 0.05) using SPSS software (20^th^edition). In addition, normality and variance were considered for statistical significance. Different letters presented in table and figures indicate significant differences between the mean ± SD of treatments (n = 3) at P < 0.05 significance level, where applicable. Further, GraphPad Prism 6 software was used to prepare graphical figures.

## 3 Results

### 3.1 Seed texture and pH

**Fig 1:**
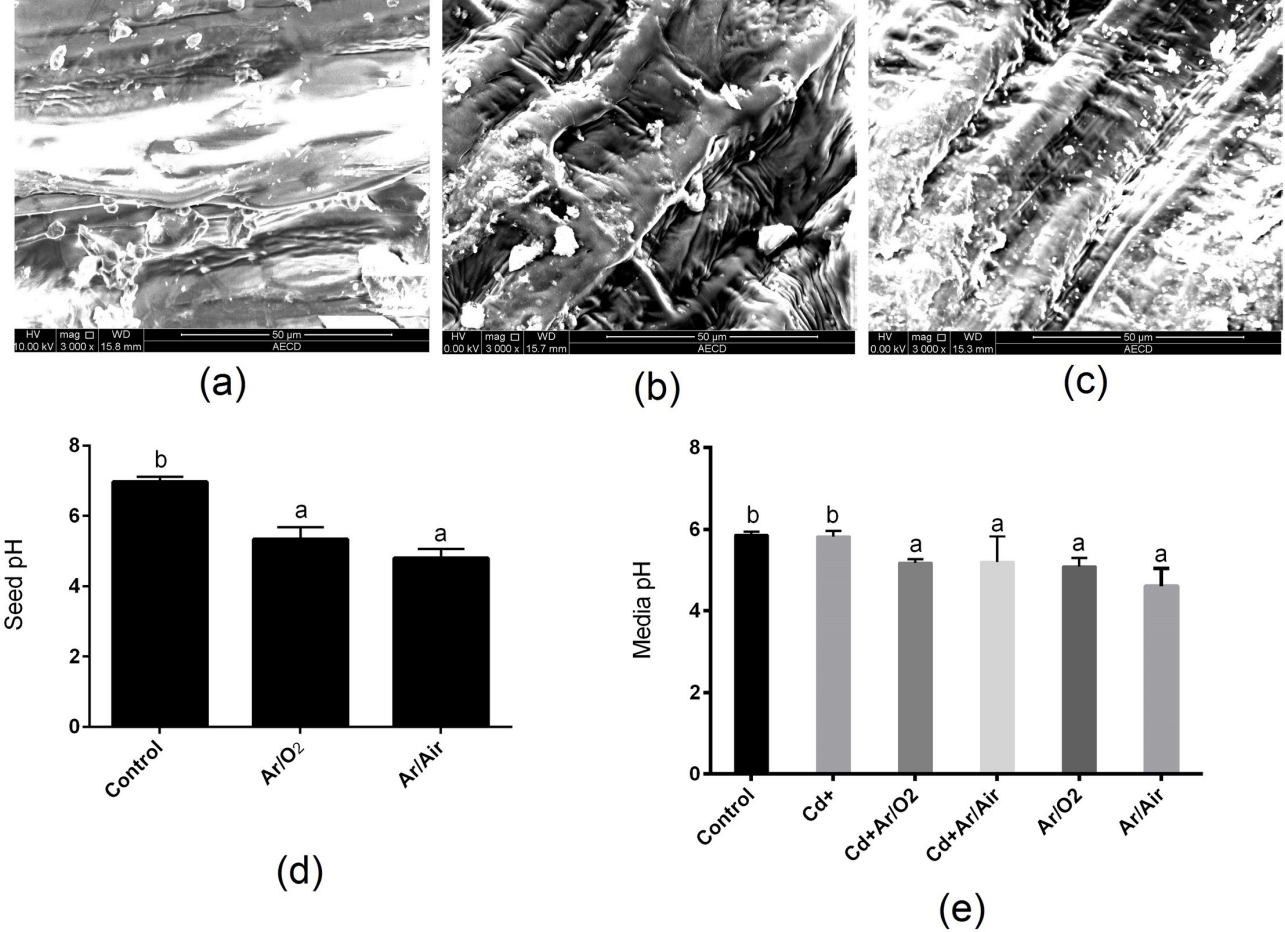
**SEM images (Scale bar is 50 μm.) of wheat seed surface treated for 90 s with (a) control (no plasma), (b) Ar/O2 and (c) Ar/Air (d) seed pH and (e) media pH.** Different letters indicate significant differences between mean ± SD of treatments (n = 3) at P < 0.05 significance level, where applicable.

The surface of seeds was much rougher due to both Ar/O_2_ and Ar/Air plasma treatment (Fig 1B and 1C) compared to those of control seeds (Fig 1A). Further, the seed coat became eroded and chapped by plasma treatment (Fig 1B and 1C). The pH of the seeds was significantly reduced due to Ar/O_2_ and Ar/Air plasma compared with untreated seeds (Fig 1D). In addition, the pH of the solution culture containing plants with or without Cd supplementation derived from Ar/O_2_ and Ar/Air plasma was significantly decreased compared to the solution with plants grown from untreated seeds (Fig 1E).

### 3.2 Morpho-physiological characteristics of wheat seedlings

**Fig 2:**
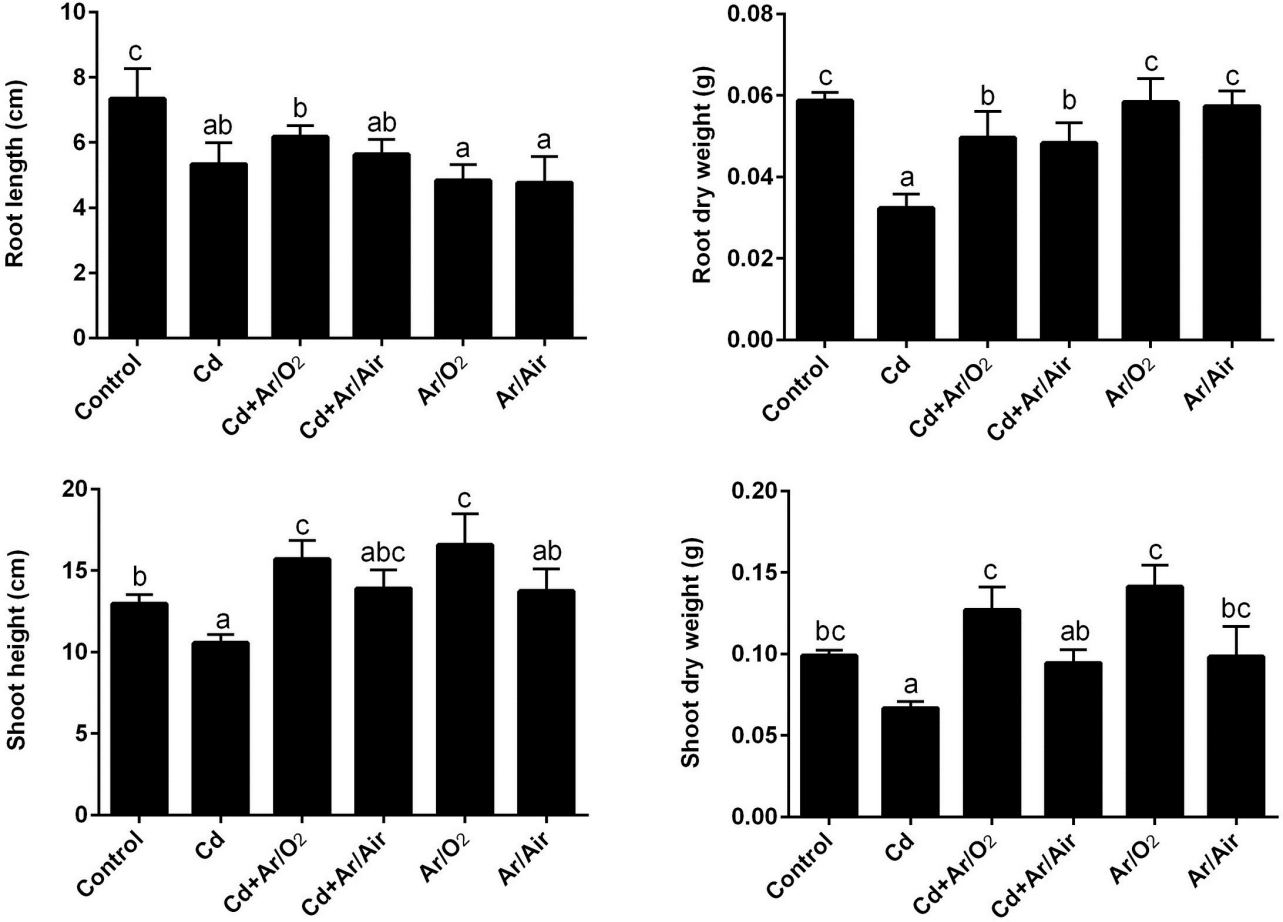
Morphological characteristics of wheat seedlings cultivated from seeds treated with Ar/O_2_ and Ar/Air plasmas in the absence or presence of Cd. Different letters indicate significant differences between mean ± SD of treatments (n = 3) at P < 0.05 significance level.

The presence of Cd in the hydroponic culture resulted in a significant decrease in the length of the root, dry weight, and height of the shoot and dry weight of wheat compared to untreated controls (Fig 2). Although the seeds treated with Ar/O_2_ and Ar/Air caused no significant improvement in the length of the root, the root dry weight increased significantly on Cd stress compared to the plants treated exclusively with Cd (Fig 2). Further, plantsgrown from Ar/O_2_ and Ar/Air seeds showed a significant decrease in root length but not root dry weight compared to controls (Fig 2). In addition, both shoot height and shoot dry weight significantly increased in plants treated with Ar/O_2_ plasma compared to the plants supplemented with Cd (Fig 2). Plants treated with Ar/Air plasma, however, showed no changes in shoot characteristics. Further, Ar/O_2,_ but not Ar/Air, treated alone showed a significant increase in shoot height compared to controls (Fig 2).

### 3.3 Chlorophyll (*a* and *b*) and Cd concentration

**Fig 3:**
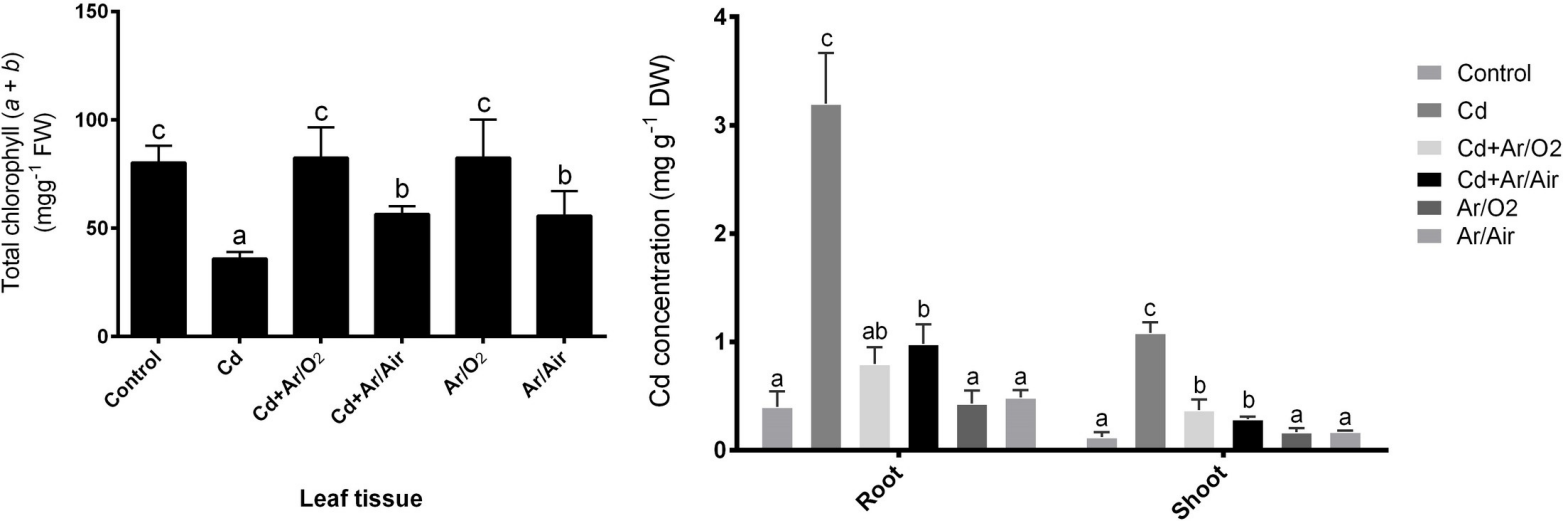
Total chlorophyll (a and b) and Cd concentration in wheat seedlings cultivated from seeds treated with Ar/O_2_ and Ar/Air plasmas in the absence or presence of Cd. Different letters indicate significant differences between mean ± SD of treatments (n = 3) at P < 0.05 significance level.

Wheat leaves showed a significant decline in total chlorophyll concentration (*a* and *b*) due to Cd supplementation compared with non-treated controls (Fig 3). However, plants grown from seeds treated with Ar/O_2_ and Ar/Air plasma in the presence or absence of Cd showed a significant increase in the total concentration of chlorophyll compared to plants stressed by Cd. In addition, the root and shoot Cd significantly increased in response to Cd compared with non-treated controls (Fig 3). However, the root and shoot of plants derived from seeds of Ar/O_2_ and Ar/Air showed a significant reduction in the concentration of Cd compared to plants stressed by Cd. Plants treated with plasma Ar/O_2_ and Ar/Air grown without Cd showed the same concentration of Cd in leaves as untreated controls (Fig 3).

### 3.4 Biochemical features

**Fig 4:**
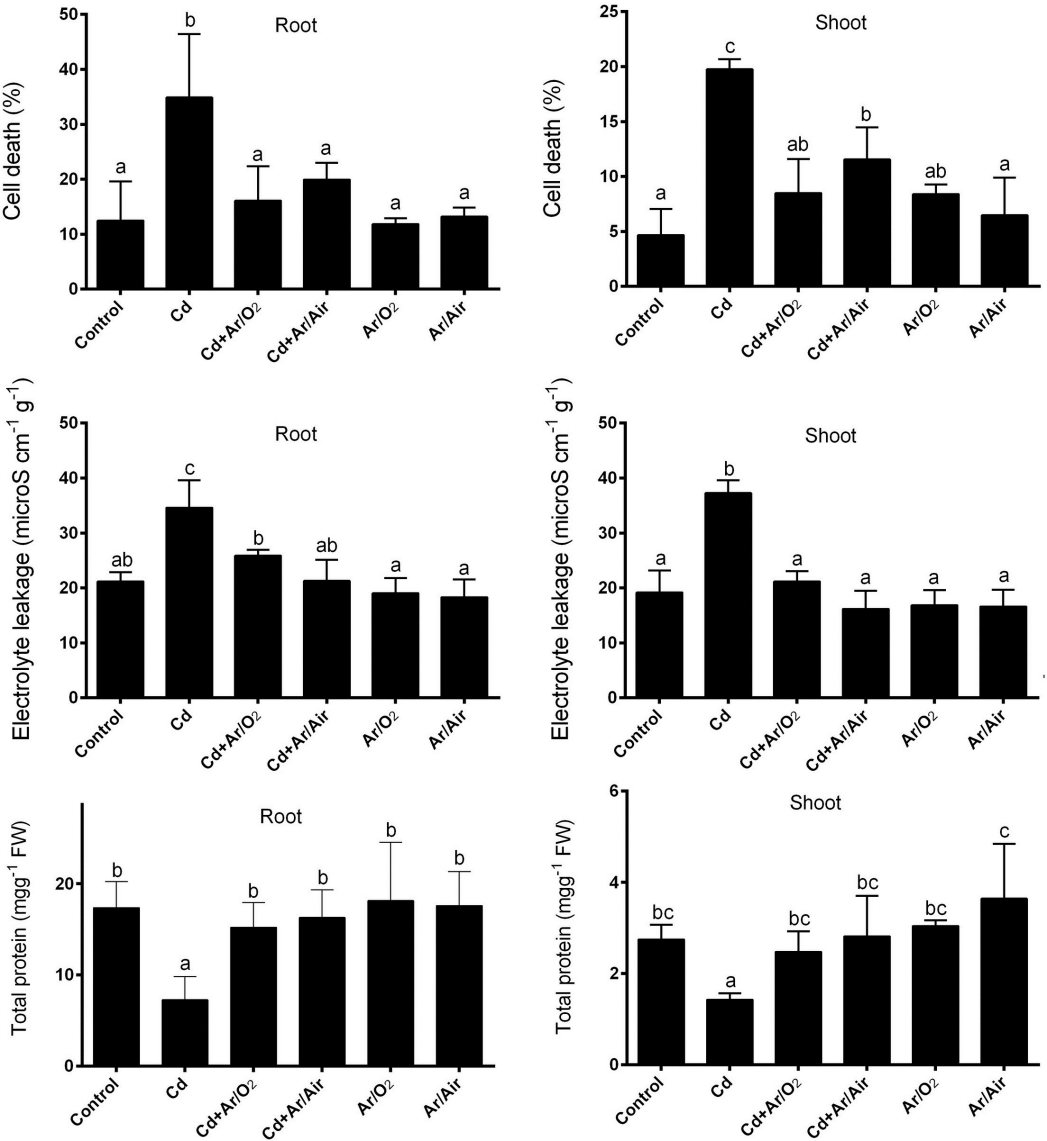
Biochemical characteristics in root and shoot of wheat seedlings cultivated from seeds treated with Ar/O_2_ and Ar/Air plasmas in the absence or presence of Cd. Different letters indicate significant differences between mean ± SD of treatments (n = 3) at P < 0.05 significance level.

Cell death (%) and electrolyte leakage were significantly increased in response to Cd in root and shoot compared with controls (Fig 4). However, these stress indicators showed a significant decline in plants derived from Ar/O_2_ and Ar/Air plasma compared with Cd-treated plants. Cell death (%) and electrolyte leakage showed similar results in plants treated with Ar/O_2_ and Ar/Air plasma without Cd supplementation to that of controls (Fig 4). In addition, the presence of Cd in the solution culture showed a significant decrease in total soluble protein in response to Cd in both roots and shoot compared to the non-treated controls (Fig 4). Compared to Cd-stressed plants, the total soluble protein increased significantly in root and shoot in the presence or absence of Cd in plants derived from Ar/O_2_ and Ar/Air plasma (Fig 4).

### 3.5 Changes in signaling molecules (H_2_O_2_ and NO)

**Fig 5:**
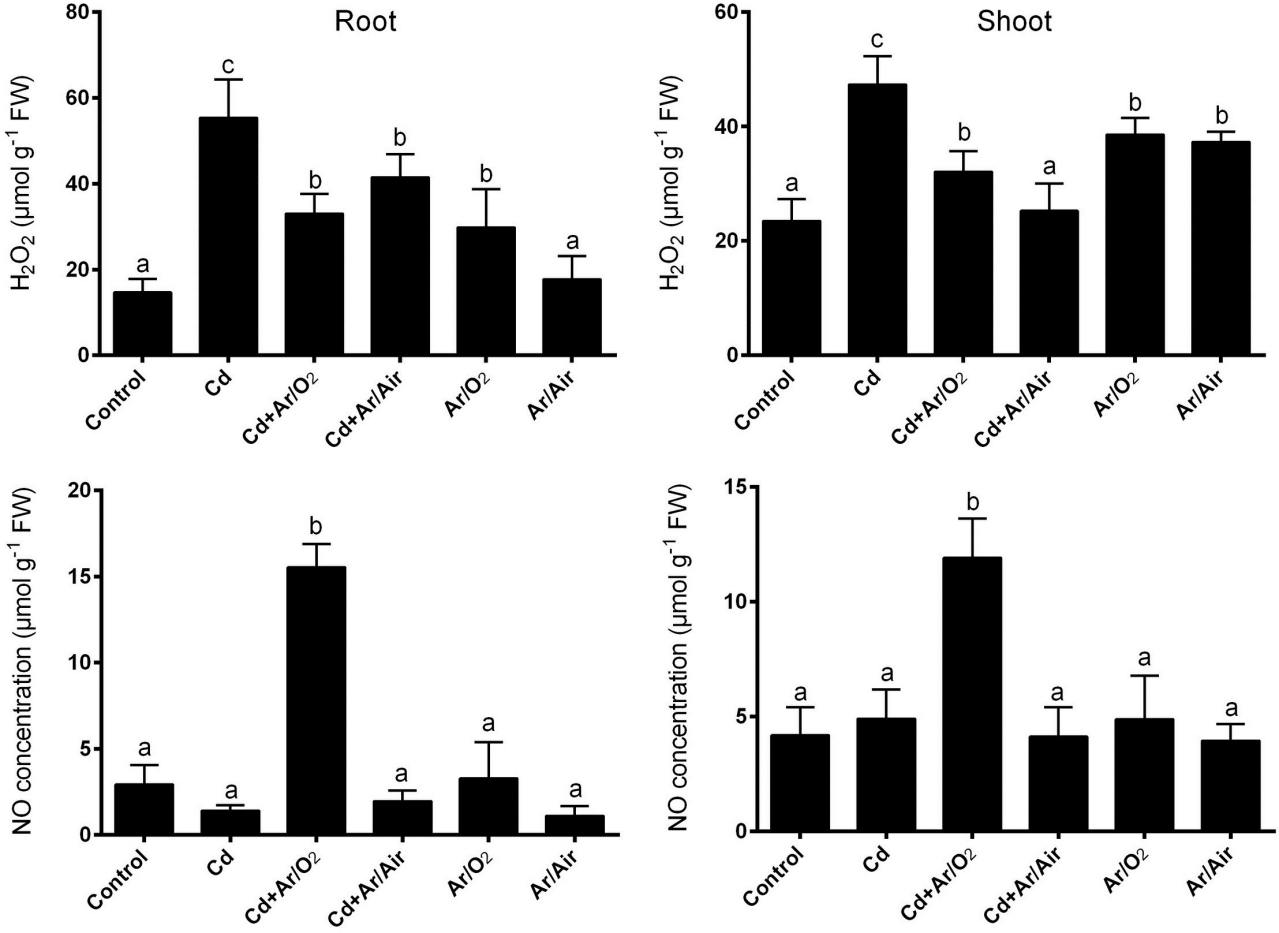
H_2_O_2_ and NO concentration in root and shoot of wheat seedlings cultivated from seeds treated with Ar/O_2_ and Ar/Air plasmas in the absence or presence of Cd. Different letters indicate significant differences between mean ± SD of treatments (n = 3) at P < 0.05 significance level.

Compared to controls, the concentration of H_2_O_2_ in root and shoot increased significantly in the presence of Cd (Fig 5). However, seeds treated with Ar/O_2_ and Ar/Air caused a significant reduction in H_2_O_2_ in the root or shoot compared to plants grown in the presence of Cd (Fig 5). In addition, NO showed a significant increase in both roots and shooting only when seeds were treated with Ar/O_2_ plasma grown in the presence of Cd compared to other treatments (Fig 5).

### 3.6 Changes in antioxidant enzymes

**Fig 6:**
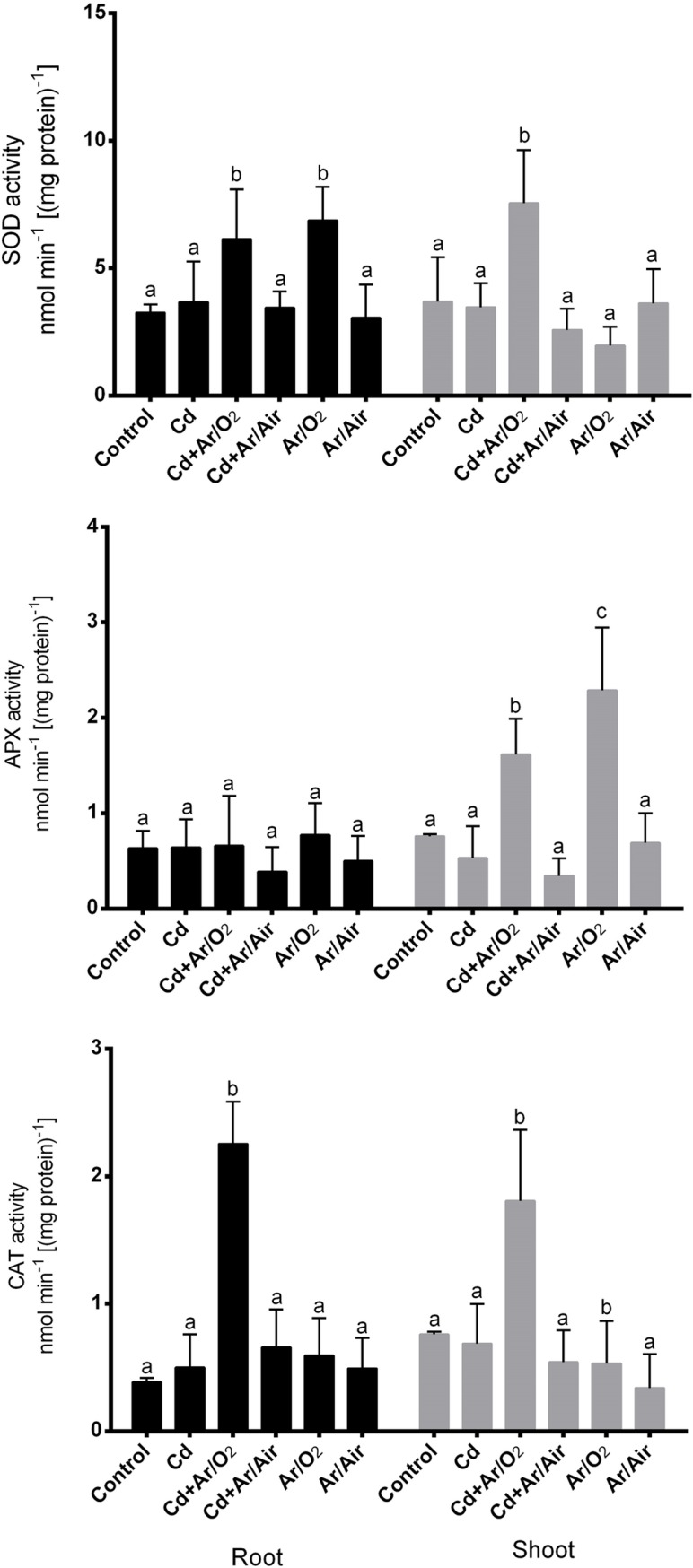
Activities of antioxidant enzymes (SOD, APX and CAT) in root and shoot of wheat seedlings cultivated from seeds treated with Ar/O_2_ and Ar/Air plasmas in the absence or presence of Cd. Different letters indicate significant differences between mean ± SD of treatments (n = 3) at P < 0.05 significance level.

SOD activity showed no significant changes compared to controls due to Cd stress in the root or shoot (Fig 6). In addition, plants grown in the presence or absence of Cd derived from plasma Ar/O_2_ showed a significant increase in SOD activity only in roots compared to plants stressed by Cd (Fig 6). However, in comparison with other treatments, Ar/Air plasma showed no significant effect on SOD activity in the root or shoot (Fig 6). There was no change in the roots of the APX activity in the treatments. In addition, plasma-treated Ar/O_2_ plants grown with or without Cd showed a significant increase in the activity of APX in shoot compared to plants not treated with plasma grown in the presence or absence of Cd (Fig 6). Compared to other treatments, Ar / Air plasma showed no significant changes in APX activity in the root or shoot (Fig 6). CAT activity showed no significant alteration in either root or shoot under Cd supplementation compared to controls (Fig 6). However, CAT activity was significantly increased in plants derived from Ar/O_2_ plasma in the presence of Cd in comparison with the treatments (Fig 6). Compared to other treatments, Ar/Air plasma has shown no significant effect on CAT activity in the root or shoot.

### 3.7 Expression of candidate genes

**Fig 7:**
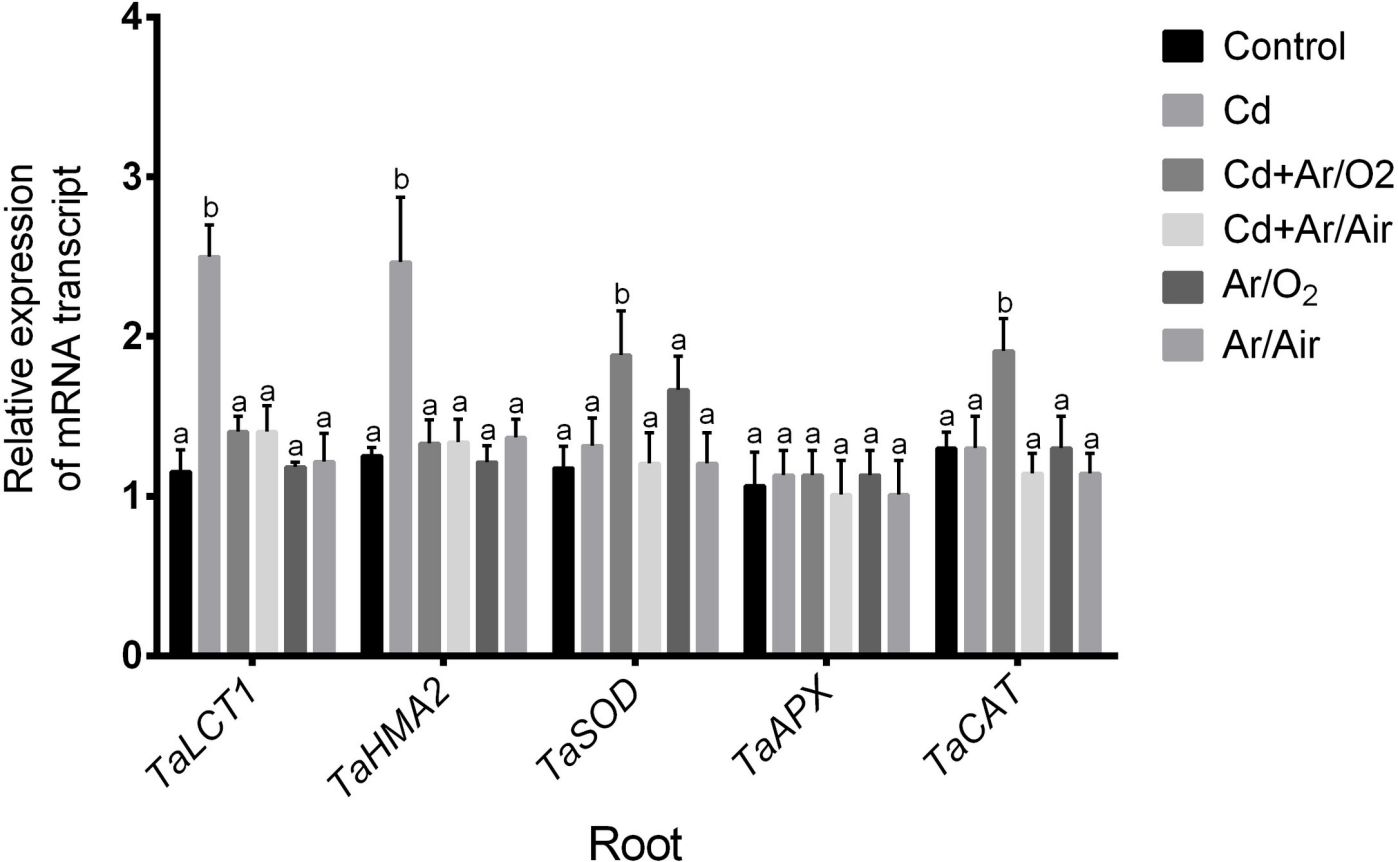
Quantitative expression analysis of *TaLCT1*, *TaHMA2*,*TaSOD*, *TaAPX* and *TaCAT* in root and shoot of wheat seedlings cultivated from seeds treated with Ar/O_2_ and Ar/Air plasmas in the absence or presence of Cd. Different letters indicate significant differences between mean ± SD of treatments (n = 3) at P < 0.05 significance level.

Major genes (*TaLCT1* and *TaHMA2*) responsible for Cd uptake in roots of wheat were significantly upregulated under Cd stress compared to untreated controls (Fig 7). However, application of Ar/O_2_ and Ar/Air plasma in seeds caused a significant decrease in *TaLCT1* and *TaHMA2* expression in roots either grown with or without Cd in comparison with Cd-stressed plants (Fig 7). Further, the expression *TaSOD* and *TaCAT* showed no significant change due to Cd stress; however, the application of Ar/O_2_ plasma in seeds caused a significant increase in roots of wheat with Cd stress compared with any other treatments (Fig 7). Further, our real-time PCR analysis demonstrated no significant change in *TaAPX* expression among the treatment. However, Ar/Air plasma did not show any effect on the expression of these transcripts (Fig 7).

### 3.8 Reciprocal grafting of plants

Root length, root dry weight, and shoot dry weight significantly decreased in Type 1 grafts compared with type 2 grafts (Table 1). Further, type 3, 5 and 6 grafted plants showed a significant increase in these morphological features compared with type 2 and 4. With the exception of a significant increase in type 6, the shoot length showed no changes in any type of grafted plants (Table 1). In the biochemical analysis, H_2_O_2_was significantly increased in root and shoot in type 2 and 4 in comparison with type 1, 3, 5 and 6 (Table 1). In addition, NO level was significantly increased in root and shoot of type 3, 5 and 6 grafts compared with type 1, 2, 4 (Table 1).

**Table 1:** Morphological features, H_2_O_2_ and NO concentration in different combinations of grafted plants supplemented with or without Cd grown from the seeds treated with Ar/O_2_plasma.

Features	Root length (cm)	Root DW (g)	Shoot length (cm)	Shoot DW (g)	H_2_O_2_ (μmol g^-1^ FW)		NO (μmol g^-1^ FW)	
					root	shoot	root	shoot
Type 1	3.9±0.40^bc^	0.028±0.002^b^	5.0±0.05^ab^	0.044±0.002^bc^	19.3±5.1^a^	19.9±3.8^a^	4.9±0.26^a^	5.2±0.11^a^
Type 2	3.2±0.36^a^	0.021±0.001^a^	4.1±0.32^a^	0.029±0.002^a^	37.3±9.6^b^	43.6±3.6^b^	4.9±0.75^a^	4.5±0.35^a^
Type 3	4.1±0.23^c^	0.036±0.008^c^	4.9±0.75^ab^	0.040±0.005^bc^	19.3±1.1^a^	21.5±3.8^a^	13.1±1.87^b^	13.5±2.01^b^
Type 4	3.3±0.41^ab^	0.020±0.001^a^	4.1±0.28^a^	0.027±0.003^a^	41.1±10.7_b_	42.9±2.8^b^	4.6±0.79^a^	4.5±0.20^a^
Type 5	4.5±0.5^c^	0.031±0.003^bc^	4.4±0.47^a^	0.037±0.002^b^	17.2±3.0^a^	18.9±4.4^a^	13.6±2.13^b^	14.9±2.5^b^
Type 6	4.6±0.32^c^	0.035±0.001^bc^	5.6±0.57^b^	0.045±0.006^c^	19.1±3.1^a^	21.7±5.5^a^	15.6±0.55^b^	14.13±1.41^b^

Different letters in each column indicate significant differences between the mean ± SD of treatments (n = 3) at P < 0.05 significance level.

type 1: control self-grafting; type 2: control self grafting under Cd stress; type 3: Ar/O_2_ self-grafting under Cd.; type 4: control rootstock + Ar/O_2_ scion under Cd stress; type 5: Ar/O_2_ rootstock + control scion under Cd stress; type 6: Ar/O_2_ self-grafting.

## 4 Discussion

Reducing the uptake of environmental toxins into wheat is necessary to reduce the hazards that are associated with health hazards for human populations. Recently, plasma technology drew attention for its effect on agronomic improvement in crop plants. Our study reveals the effectiveness and mechanisms of LPDBD plasma to alleviate Cd toxicity in wheat plants.

In this study, LPDBD plasma caused a rough texture in the seed surface. Changes in seed surface and germination enhancement due to plasma technology were also previously reported [19, 30, 31]. In addition, LPDBD plasma treatments produced different functional reactive species. In our study, both Ar/O_2_ and Ar/Air plasma treatments decreased the pH of seed and the wheat cultivation media compared to the untreated conditions. The decrease of pH due to plasma was also reported in radish, tomato, and sweet pepper [32]. Changes in the pH are possibly due to the interaction of plasma with complex macromolecules [33]. Further, plasma discharge causes the acidification of water along with reactive nitrogen species such as NO, NO_2,_ and NO_3_ [15]. In a previous study, maintenance of low pH in growth conditions caused the suppression of Cd uptake in several plant species [34]. In this present study, inhibition of Cd uptake might be associated with the increased competition of Cd with hydrogen ion released by plasma species. Cd stress caused a severe decline in morphological characteristics along with leaf chlorophyll concentration in wheat plants. In this study, the seed treatment with Ar/O_2_ and Ar/Air plasma significantly mitigated these morpho-physiological retardations in wheat induced by Cd stress. In comparison, Ar/O_2_ proved to be more efficient in wheat growth than Ar/Air plasma. Interestingly, higher shoot height was observed due to Ar/O_2_ even though plants were grown with Cd stress. It might be associated with optimum H_2_O_2_ produced by plasma treatments as previously reported in wheat [19]. As anticipated, Cd level in root and shoot dramatically increased subjected to Cd treatment in wheat plants. Interestingly, Cd concentration in root and shoot showed a significant decrease in plants cultivated from Ar/O_2_ and Ar/Air treated seeds. Toxic metal uptake in plants is closely linked with the pH. Studies showed that uptake of Cd by ryegrass, water grass, and lettuce significantly decreased due to acidification of solution culture [17]. However, the effect of pH on Cd uptake is generally species dependent. In rice, the total absorption of Cd was increased with a reduction in pH and an increase in redox potential [33].Being consistent with the physiological observations, the expression pattern of Cd transporters (*LCT1* and *HMA2*) was significantly downregulated in roots of wheat plants grown from Ar/O_2_ and Ar/Air treated seeds. Our findings reveal that plasma technology could be a promising eco-friendly strategy of Cd remediation through pH adjustment, depending on the tolerance level of low pH in plants.

We further studied the changes of a few stress indications in response to plasma treatment. We found that Cd-induced deleterious effect on cellular and protein features notably improved due to Ar/O_2_ and Ar/Air treatments in seed before germination in wheat. It might concur with the reduced Cd uptake and translocation in wheat cultivated from plasma treated seeds. Generally, Cd stress interacts with cellular events resulting in the cell membrane and protein degradations in plants [3, 35].

Stress-induced signaling is crucial for abiotic stress tolerance in plants. H_2_O_2_ in plant physiology is usually considered as toxic molecules at high concentration [36]. However, H_2_O_2_ plays critical roles in several processes including development and stress responses in plants [19, 37, 38]. In addition, the regulation of H_2_O_2_ is often associated with the activities of ROS scavenging enzymes. Due to Ar/O_2_ and Ar/Air plasma treatments, the elevation of H_2_O_2_ in root and shoot was significantly reduced in this study. This reduction in H_2_O_2_ was further supported by the increased activity of SOD and CAT activities along with its corresponding *TaSOD* and *TaCAT* genes in the plants cultivated from plasma treated seeds. This implies that plasma discharge is associated with the elevated ROS scavenging activity, which causes indirect inhibition of H_2_O_2_ in wheat tissues. We further investigated the involvement of NO which is known to play an important task as signaling messenger in abiotic stress tolerance in plants. Among the two different plasma treatments, Ar/O_2_ showed a sharp increase in root and shoot characteristics in wheat plants only when the plants were supplemented with Cd stress. Our reciprocal grafting experiment revealed that grafts combined with the Ar/O_2_-derived rootstock either with Ar/O_2_ or control scion exhibited morphological characteristics, H_2_O_2_ and NO concentration similar to those of plants tolerant to Cd stress cultivated from Ar/O_2_ treated seeds. It does suggest that mechanisms governing LPDBD plasma mediated alleviation of Cd toxicity in wheat is originated in roots and is possibly governed by NO signaling molecule.

## 5 Conclusion

This work reveals that seeds treated with LPDBD are effective for morphological and biochemical improvement as well as Cd detoxification in wheat plants. Our results suggest that LPDBD plasma, especially Ar/O_2,_ induces pH reduction resulting in less bioavailability of Cd for plants. Further, LPDBD plasma-derived species produced elevated antioxidant enzymes and their candidate genes, which in turn protect plants from Cd-induced oxidative damage. Grafting data suggest that alleviation of Cd toxicity due to LPDBD plasma possibly originates in root and drive the mechanisms through NO signaling. Overall, our findings reveal that plasma technology can alleviate the damages and reduce the growth retardation in Cd-stressed wheat plants. These outcomes can be further applied to establish an eco-friendly strategy for the prevention of heavy metal toxicity in other crop plants to meet the global need for food safety.

## Supporting information

S1 Table List of primers used in qPCR experiments. (DOCX)

S1 FigSchematic diagram of (a) LPDBD plasma for wheat treatment with Ar/O2 and Ar/Air gases, (b) V-I waveform of Ar/Air LPDBD plasma measured at applied voltage 5kV and electrode spacing 60 mm and (c) Emitted spectrum from Ar/O2 and Ar/Air LPDBD plasmas at applied voltage 5kV and electrode spacing 60 mm.(TIF)
