# Supplementary material for: Reduction of cadmium toxicity in wheat through plasma technology
Source: PLoS One. 2019 Apr 1;14(4):e0214509. doi: 10.1371/journal.pone.0214509 (PMC6443147; doi:10.1371/journal.pone.0214509)
Supplement: S1 Table — (DOCX) [file pone.0214509.s001.docx]

| Gene Name | Accession number | Primer sequences |
| --- | --- | --- |
| *TaActin* | AY212324 | Forward: GAATCCATGAGACCACCTAC  Reverse: AATCCAGACACTGTACTTCC |
| *TaLCT1* | AF015523.2 | Forward: ATCTTTGGTTTCGCTGTGCT  Reverse: GACGATGTTCATGCTGAGGA |
| *TaHMA2* | JN113581.1 | Forward: CAGATCGGTAAGCAAGCACA  Reverse: GAAAGCGAGCAGTTTTCACC |
| *TaSOD* | AF439787.2 | Forward: CGAAGATTCCATTTCCCAGA  Reverse: TCGAGGATATCGGTGAAAGC |
| *TaAPX* | AB559521.1 | Forward: TCCATCCAACCAAACCCGGAAA  Reverse: TGCCAATGTCCTTCTGTTCCCA |
| *TaCAT* | S81897.1 | Forward: TTGGACCAGGCTTTATGGTC  Reverse: CCCTGTCACCACTCCAAGAT |
