# Supplementary figures and images for: Reduction of cadmium toxicity in wheat through plasma technology
Source: PLoS One. 2019 Apr 1;14(4):e0214509. doi: 10.1371/journal.pone.0214509 (PMC6443147; doi:10.1371/journal.pone.0214509)

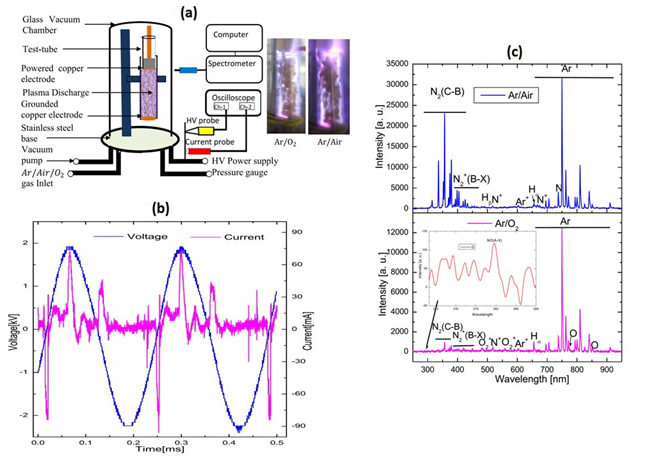

Supplement: S1 Fig — Schematic diagram of (a) LPDBD plasma for wheat treatment with Ar/O2 and Ar/Air gases, (b) V-I waveform of Ar/Air LPDBD plasma measured at applied voltage 5kV and electrode spacing 60 mm and (c) Emitted spectrum from Ar/O2 and Ar/Air LPDBD plasmas at applied voltage 5kV and electrode spacing 60 mm. (TIF) [file pone.0214509.s002.tif]
